# In Situ Characterization of the Oxidation Behavior of Carbonate-Based Electrolytes for Lithium-Ion Batteries by Scanning Electrochemical Microscopy

**DOI:** 10.1021/acselectrochem.4c00106

**Published:** 2024-12-04

**Authors:** Rong He, Liam McDonough, Liam Seitz, Wenhan Ou, Samuel D. Marks, Rafael Ferreira de Menezes, Elizabeth Allan-Cole, Hongmei Luo, Michael F. Toney, Kayla G. Sprenger, Meng Zhou, Robert C. Tenent

**Affiliations:** † National Renewable Energy Laboratory, Golden, Colorado 80401, United States; ‡ Department of Chemical and Materials Engineering, New Mexico State University, Las Cruces, New Mexico 88003, United States; § Materials Science and Engineering, University of Colorado Boulder, Boulder, Colorado 80303, United States; ∥ Department of Chemical and Biological Engineering, University of Colorado Boulder, Boulder, Colorado 80303, United States; ⊥ Renewable and Sustainable Energy Institute, University of Colorado at Boulder, Boulder, Colorado 80309, United States

**Keywords:** Scanning electrochemical microscopy, electrolyte decomposition, cathode-electrolyte interphase, lithium-ion batteries

## Abstract

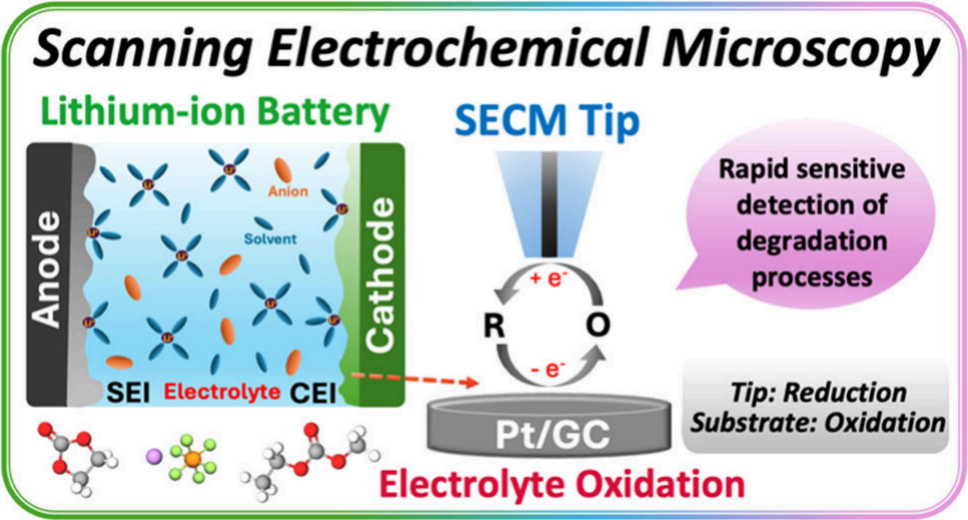

Lithium-ion batteries (LIBs) have been widely employed as energy storage devices in portable electronics and electric vehicles. Many processes occurring at the electrode/electrolyte interphases lead to performance degradation over time and yet remain poorly understood. We demonstrate new methods based on scanning electrochemical microscopy (SECM) to characterize LIB electrolyte oxidation, which is a key process occurring at the cathode/electrolyte interphase. Our technique leverages a combination of feedback mode and generation/collection mode SECM to provide an electrochemical reversal technique like commonly used cyclic voltammetry or rotating-ring disk electrode methods but one potentially more easily applicable at shorter time scales. We use our method to probe the oxidation of LIB electrolyte components at nonintercalating electrodes including Pt and glassy carbon. We study the oxidation of a common commercially used electrolyte, 1.2 M LiPF_6_ 30% ethylene carbonate and 70% ethyl methyl carbonate electrolyte (LP58), as well as formulations using only the individual carbonates. Our results indicate that all electrolyte formulations characterized oxidize through multiple processes that are detected at distinct voltages. Some processes generate soluble and reducible products that are detected by the SECM tip electrode and may be consistent with deprotonation of carbonate solvents. However, several other oxidation processes do not appear to generate soluble and reducible species and may be connected to the formation of either nonelectroactive products or of processes only occurring on the substrate electrode surface. This work provides information about the electrochemical oxidation of commonly used carbonate electrolytes for comparison to studies involving potentially more complex processes in the presence of LIB cathode materials.

## Introduction

Electrochemical energy storage systems are considered sustainable solutions to reduce our carbon footprint and help manage the current global energy crisis.^1,2^ Lithium-ion batteries (LIBs) have been widely utilized as power sources for various devices such as portable electronics and electric vehicles, owing to their high energy density and rechargeable features.^3−5^ During cycling of LIBs, decomposition products generate layers on the electrodes, resulting from reactions occurring at the electrode/electrolyte interfaces (EEI).^6^ The layers are referred to as “interphases” as they typically involve both solution and solid components. The interphase formed on the negative electrode (anode) was the first identified and is commonly referred to the “solid electrolyte interphase (SEI)”.^7^ The SEI has been widely explored and is typically composed of inorganic compounds as well as degraded electrolytes and solvents that can act as a passivation layer to protect anodes from further degradation.^8^ Similarly, the interphase generated at the cathode during cycling is referred to the “cathode-electrolyte interphase (CEI)” and has been much less studied.^9^ Some processes occurring at the CEI are known to lead to products that diffuse through the cell and drive failure. However, most of these processes are poorly understood. Mechanistic insight into the formation of the CEI and resulting interphase layer properties is essential to develop rational strategies to enhance the LIB cycle life and safety.

CEI formation appears to occur through multiple overlapping processes, leading to degradation of both cathode as well as electrolyte materials. Cathode degradation mechanisms include transition metal dissolution and structural reconstruction with reaction pathways that likely vary over different types of cathodes and electrolyte chemistries.^10−13^ Electrolyte degradation occurs through the oxidation of electrolyte solvents or salt components. Recent work has shown that degradation of LIB electrolytes may proceed through multiple potential oxidation mechanisms.^14,15^ Electrochemical oxidation involves direct transfer of electrons from the electrolyte to the electrode. In addition to the direct electron transfer route, chemical oxidation processes have been proposed that may involve attack of electrolyte components by reactive species generated from cathode materials at high voltage.^16,17^ Recent studies report that O_2_ in the form of a singlet excited state may be released from metal oxide cathodes and react with common carbonate electrolyte solvents during cycling.^18−20^ This singlet oxygen pathway has recently been reconsidered by the Persson and McCloskey groups using density functional theory (DFT).^21^ This team has suggested an alternative mechanism in which ethylene carbonate (EC) reacts with superoxide (O_2_^–^) and/or peroxide (O_2_^2–^) anions.^21^

Varied techniques have been applied to explore electrolyte oxidation behavior and CEI formation.^22,23^ For example, Nagarajan et al. utilized high-resolution transmission electron microscopy (HR-TEM) to visualize the existence of conformal CEI layers formed on the LiNi_0.33_Mn_0.33_Co_0.33_O_2_ (NMC333) surface after cycling in a potential region of 2.8–4.3 V at 100 °C.^24^ In addition, Kim et al. confirmed the formation of an unstable CEI layer on NMC532 by secondary-ion mass spectrometry (SIMS).^22^ They reported that positive charge transfer from the cathode bulk to the surface layer was determined by the energy level position and band bending, indicating a loss of Li^+^ from the cathode. An unstable CEI layer was formed due to the accumulation of deintercalated Li^+^ ions, which eventually dissolve in the electrolyte, resulting in capacity fade. These findings indicate that the formation of the CEI is a dynamic process and that CEI properties may evolve over time. Therefore, in situ characterization techniques are vital for better understanding the formation and evolution of the CEI.

A variety of electrochemical methods have been used to study CEI formation as well as coupled electrolyte oxidation processes.^25,26^ Direct electrochemical oxidation of carbonate-based electrolytes was studied by Azcarate et al. using cyclic voltammetry.^27^ This work demonstrated that carbonate electrolyte oxidation likely occurs through several steps, including the formation of organic polycarbonate-rich films. Hatsukade et al. developed a rotating ring-disc electrode (RRDE) voltammetry method to assess electrolyte oxidation processes in situ.^28^ RRDE voltammograms for the oxidation of carbonate electrolytes containing 1.2 M LiClO_4_ were collected at a Pt (111) disk electrode and showed electrolyte oxidation at high potentials with a soluble reducible product detected (at the ring electrode) at ∼3.16 V. This signal was linked to the reduction of protons generated from oxidation of the carbonate solvents. While these papers demonstrated the complexity of carbonate electrolyte oxidation and the identification of at least one product, they were not able to link the formation of products to specific steps in the oxidation process.

**Figure 1:**
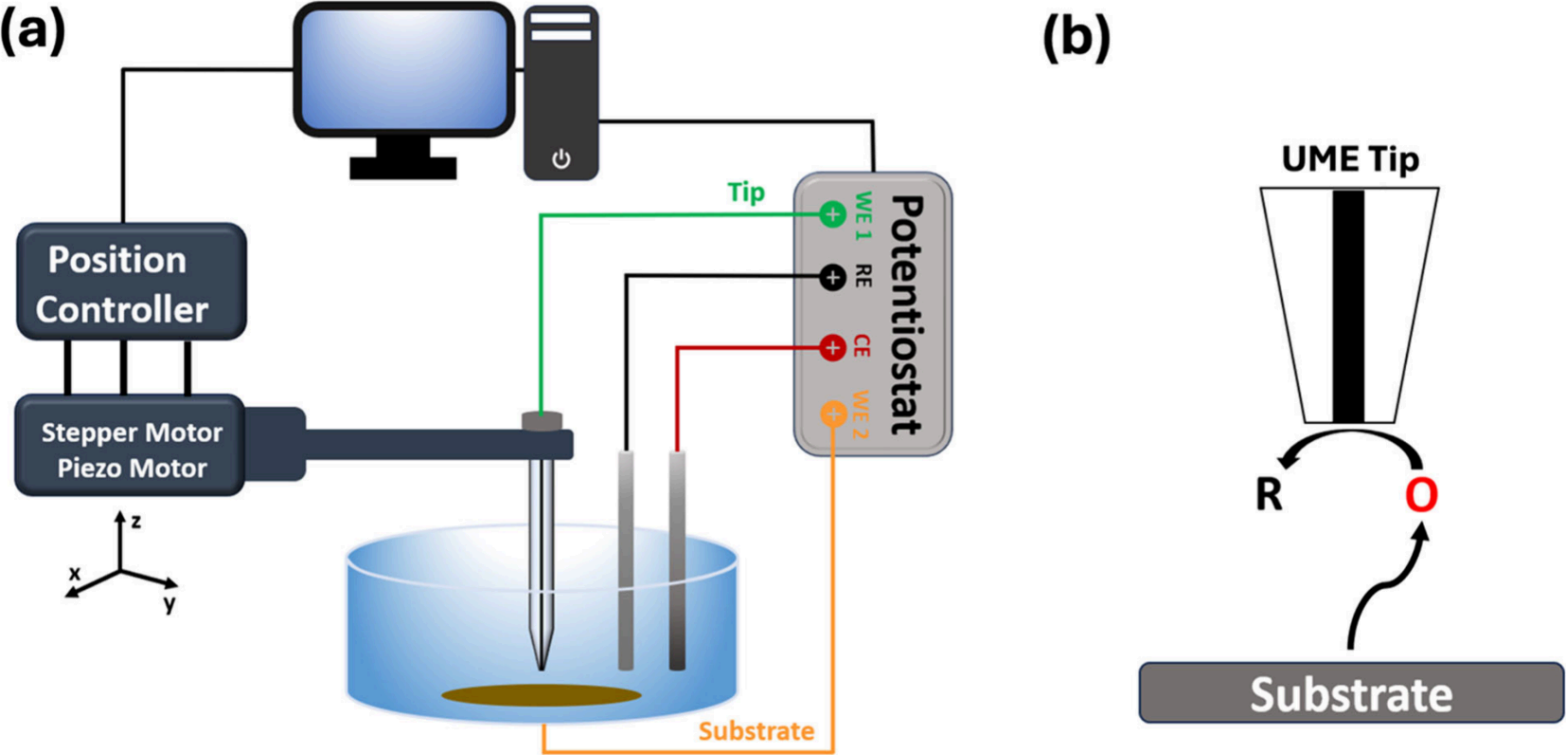
Schematic illustration of (a) the SECM setup and (b) substrate-generation/tip-collection (SG-TC) mode.

Scanning probe microscopy techniques can enable direct characterization of interfacial processes occurring in active electrochemical devices.^29−31^ Scanning electrochemical microscopy (SECM) is a specific type of scanning probe technique that is focused on the characterization of the electrochemistry occurring at the electrode/electrolyte interface.^32^ SECM has been used to study a variety of interfacial electrochemical processes with both high spatial and temporal resolution.^8,33,34^ SECM measurements employ an ultramicroelectrode (UME) to probe the local electrochemistry occurring near a sample surface. The method can obtain chemical/electrochemical reactivity and/or topography of substrates on micro- and nanometer scales.^32,35^ A schematic of a basic SECM apparatus is shown in Figure 1a and consists of a four-electrode electrochemical cell with two working electrodes (substrate and tip) as well as counter and reference electrodes. The applied voltages at the substrate and tip (probe) electrode are controlled independently versus the same reference and counter electrodes. This allows electrochemical experiments to be conducted at the tip electrode when placed near the substrate surface, while the substrate is controlled at varying voltages. SECM has been used to study interfacial processes occurring at various anode materials, providing useful information on interfacial properties of SEIs.^36,37^ This present work is focused on better understanding similar processes occurring at the CEI. Our previous SECM studies looked broadly at CEI formation processes and indicated that the mechanism of Mn dissolution from LiMn_2_O_4_ (LMO) may depend on electrolyte composition.^13^ In particular we found that the electrochemical products of CEI formation depend heavily on the lithium salt anion studied.^13^ Following from that previous effort, this work isolates direct electrochemical oxidation of varied carbonate solvents containing commonly used LiPF_6_ salts. This is intended to provide baseline data on carbonate oxidation for comparison to earlier work as well as companion studies with model metal oxide cathodes and composite electrodes that enable potential chemical oxidation routes.

We demonstrate an SECM voltametric “generation/collection” method applied to the study of LIB electrolyte oxidation processes. Generation/collection SECM (G/C SECM) involves the generation of a product at either the tip or substrate electrode with detection (collection) at the opposite electrode. This leads to an electrochemical reversal technique like the cyclic voltammetry (CV), RRDE, or chronoamperometry (CA) methods but with some potential advantages. A schematic of the generation/collection mode is shown in Figure 1b. Short spacing between the tip and substrate electrodes allows rapid detection of species generated at the substrate at time scales that may may not be easily accessible using CV or RRDE methods. While CV methods can be pushed to short time scales at high scan rates, this often leads to complications with increased background current and, at times, the need for background subtraction to obtain interpretable data. By detecting products with the tip electrode at a fixed potential, the complications and associated background issues for CV are eliminated, leading to a more sensitive method for rapid detection of products. Furthermore, SECM feedback processes can be leveraged to detect the presence of coupled chemical reactions. Under SECM feedback mode, when a detected species is undergoing reversible electron transfer between the tip and substrate electrodes, a constant steady state current is obtained. Analysis of these steady state feedback processes has been used to characterize the kinetics of heterogeneous electron transfer as well as homogenous coupled reactions.^38,39^ SECM measurements can also be conducted while rastering the tip position over the substrate surface to “image” the evolution of electrochemically active products leaving the electrode surface. This work will focus on measurement method demonstration rather than imaging but will form a basis for the potential future development of imaging methods.

In this work, we specifically focus on using G/C SECM to study the direct electrochemical oxidation of carbonate electrolytes containing the [PF_6_^–^] anion at “nonintercalating” electrodes (Pt and glassy carbon). We initially demonstrate our method using the familiar ferrocene-ferrocenium redox couple as a model. The method is then applied to study the oxidation of LIB electrolytes. Results are compared with traditional methods such as linear sweep voltammetry (LSV), CV, and RRDE. Our results indicate that the G/C SECM method can resolve multiple overlapping oxidation processes occurring for carbonate solvents. Some of the observed oxidation processes lead to generation of products that can be reduced at the tip electrode, while some do not and may be related to surface-confined processes. Companion studies using CV and RRDE to date have not resolved these individual processes nor determined which led to soluble products and which may be surface processes. Based on this comparison, the G/C SECM approach enables a more in depth understanding of oxidation processes than has been feasible to date.

## Experimental Section

### Chemicals

LP58 [1.2 M lithium hexafluorophosphate in ethylene carbonate/ethyl methyl carbonate (3:7) wt %, LiPF_6_(EC:EMC)], 1.2 M LiPF_6_ in ethylene carbonate [LiPF_6_(EC)], and 1.2 M LiPF_6_ in ethyl methyl carbonate [LiPF_6_(EMC)] as electrolytes were purchased from Gotion, Inc. Ferrocene (98%) was obtained from Sigma-Aldrich. All chemicals were used as received without further purification.

### Electrochemical Measurements

The electrochemical analysis was performed on an electrochemical workstation integrated with the CHI920C SECM (CH Instruments, Inc.), where the SECM equipment was placed in an inert atmosphere glovebox that is filled with ultra-high-purity argon gas (<0.1 ppm of H_2_O and <0.1 ppm of O_2_). For a two-electrode system, a platinum (Pt, 2 mm) disk or glassy carbon (GC, 3 mm) disk electrode and Li metal were used as the working electrode (WE) and combined counter/quasireference electrode (CE/RE), respectively. Ferrocene (Fc) dissolved in the LP58 electrolyte (∼0.3 mM), referred to as LiPF_6_(EC:EMC)-Fc, was used as the model electrolyte solution for electrochemical measurements. Cyclic voltammetry was performed with a Pt or GC electrode by scanning the voltage positively from 2.8 to 3.8 V for LiPF_6_(EC:EMC)-Fc and from 2.8 to 6.0 V for other electrolytes. For the SECM cell system, a Pt or GC disk electrode was used as the substrate electrode, and Li metal as a combined counter/quasireference electrode. The Pt disk electrode was mounted in a small Teflon electrochemical cell. A Pt (10 μm) wire-embedded disk electrode was utilized as the SECM tip electrode. The Pt tip and Pt or GC substrate electrodes were polished and then washed with water under sonication before use.

### SECM Generation/Collection Mode

The experiments for studying electrolyte oxidation at Pt or GC were performed in LiPF_6_(EC:EMC), LiPF_6_(EC), and LiPF_6_(EMC). First, LiPF_6_(EC:EMC)-Fc was used as the redox mediator system to determine the distance between the UME tip and the Pt/GC substrate. After the tip reached the desired position, LiPF_6_(EC:EMC)-Fc solution was removed from the SECM cell and rinsed with fresh electrolyte LiPF_6_(EC), LiPF_6_(EMC), or LiPF_6_(EC:EMC) four times. The experimental details of tip positioning are shown in the supporting file. Background voltammograms were collected at the tip electrode following the rinsing step to confirm that all of the Fc had been removed (Figure S1). The generation/collection voltammetry measurement was conducted by scanning the substrate electrode positively from 3.5 to 6.0 V using linear sweep voltammetry with a scan rate of 50 mV s^–1^ while the UME tip was kept at a constant potential.

## Results and Discussion

**Figure 2:**
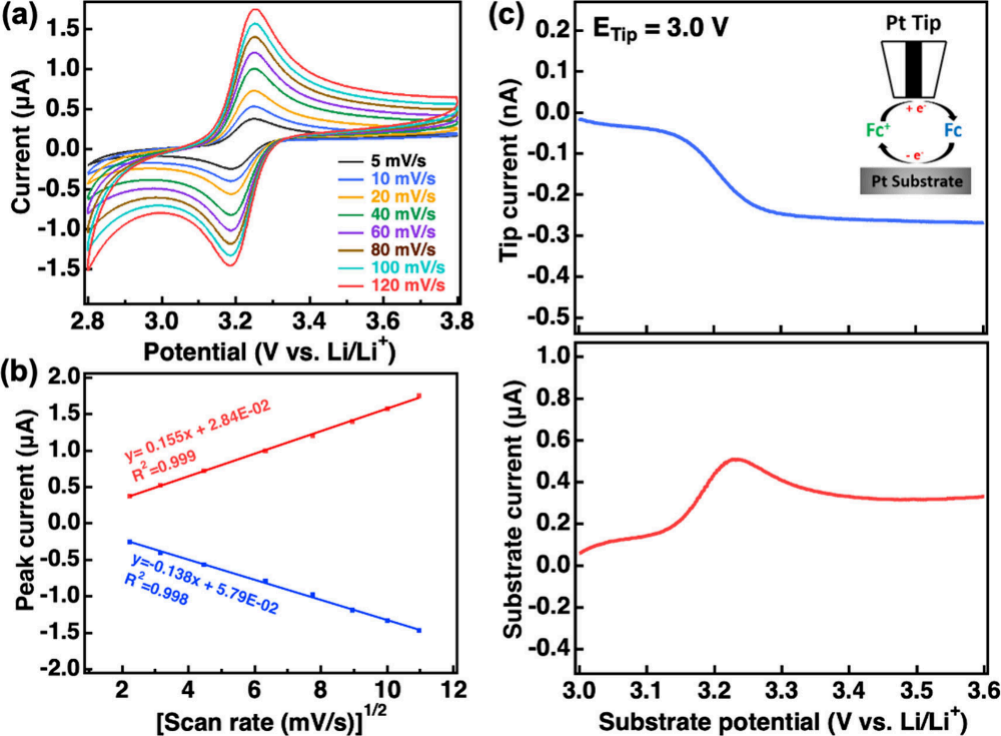
(a) Cyclic voltammetry of a Pt disk electrode (2 mm) in LiPF_6_(EC:EMC)-Fc with different scan rates; (b) cyclic voltametric peak currents as a function of the square root of the scan rate; and (c) G/C SECM measurements for a Pt electrode (2 mm) with a UME Pt tip (10 μm) held at 3.0 V in LiPF6(EC:EMC)-Fc.

To demonstrate and contrast our G/C SECM methods with traditional voltammetric measurements, an example data set was collected by using a model analyte (ferrocene) at a Pt electrode with LiPF_6_(EC:EMC) as the electrolyte. Initial electrochemical characterization of the Fc/Fc^+^ redox couple in the LiPF_6_(EC:EMC) electrolyte was conducted by using cyclic voltammetry at varied scan rates. Figure 2a shows CV data for a Pt disk electrode using the Fc/Fc^+^ redox pair in LiPF_6_(EC:EMC). Data was collected from 2.8 to 3.8 V (vs Li/Li^+^) at scan rates varying from 5 to 120 mV/s. Peaks for the oxidation and reduction of Fc occurred at about 3.24 and 3.18 V, respectively, and did not vary with scan rate. This indicates reversible electrochemical behavior of ferrocene at the Pt electrode in the LiPF_6_(EC:EMC) electrolyte.^40^ Plots of peak currents vs the square root of the scan rate are shown in Figure 2b and demonstrate a linear dependence implying that the Fc oxidation and reduction reactions are diffusion-controlled processes.^41^ This observation is consistent with other results reported in the literature.^42^

Comparison data for the oxidation and reduction of Fc were then collected using the generation/collection mode of SECM. The tip-to-substrate spacing was set at ∼10.5 μm using a 10 μm (dia.) Pt tip with a positive feedback approach technique using the Fc/Fc^+^ mediator. An example approach curve (Figure S2) and related experimental details are presented in the supporting file. G/C SECM measurements were conducted by collecting the LSV of the substrate electrode while holding the tip at a constant voltage. For the data shown in (Figure 2c), LSV data was recorded at a Pt substrate scanned from 3.0 to 3.6 V to drive the oxidation of Fc while the tip was held at a fixed voltage of 3.0 V to drive reduction of the Fc^+^ ions. The substrate LSV current response shows a peak that corresponds to Fc oxidation, as observed in earlier CV results. The current measured at the tip electrode for reduction of the ferrocenium ion shows an increase that coincides with the oxidation of Fc at the substrate and has the characteristic sigmoidal shape of a steady state response. This response arises from the continuous cycling of the Fc/Fc^+^ redox mediator between the substrate and tip electrodes in a “feedback” process as discussed earlier and is demonstrated schematically in the inset in Figure 2c. These results are consistent with the earlier CV analysis that showed reversible and diffusion-controlled behavior for Fc/Fc^+^ in the LiPF_6_(EC:EMC) electrolyte. If the ferrocenium ion was unstable in the electrolyte, deviations from this steady state response (i.e., increases or decreases in the observed current rather than a constant current) would be observed. These deviations from a steady state response can be used to detect coupled chemical reactions as well as characterize their kinetics. Kinetic characterization of these reactions is typically conducted through the analysis of current vs time transients rather than using voltametric methods. Herein, we employ a voltammetric method as an exploratory technique to identify various processes occurring during electrolyte oxidation and identify the presence of potential coupled processes. In summary, observation of a sigmoidal-shaped current response at the tip electrode in our results is consistent with reversibility of the detected process, while deviations from a steady state current imply the detected product may not be stable.

Recently, a great deal of experimental and computational efforts has focused on the oxidation of carbonate electrolytes as an important early step in CEI formation.^9,17,43^ Here we apply our G/C SECM voltammetry method to characterize the oxidation of a series of carbonate electrolytes, including the commonly used LiPF_6_(EC:EMC) system as well as the individual carbonate systems LiPF_6_(EC) and LiPF_6_(EMC) for comparison. Similarly to the study of the Fc/Fc^+^ system discussed earlier, we initially studied the oxidation process of our electrolytes using cyclic voltammetry and then compared results from the G/C SECM methods.

**Figure 3:**
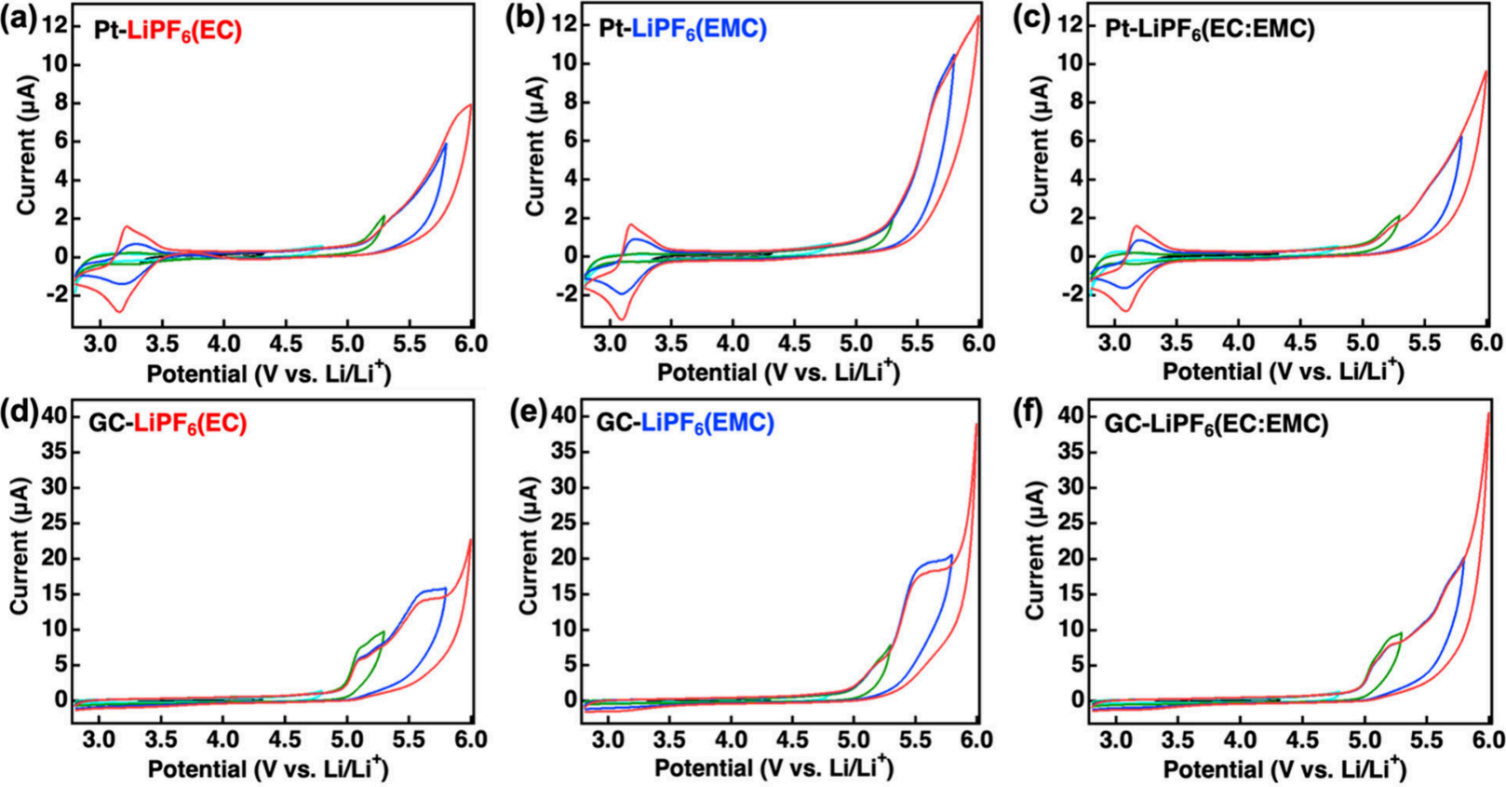
Cyclic voltammograms of (a–c) Pt electrode (2 mm) and (d–f) GC electrode (3 mm) in LiPF_6_(EC), LiPF_6_(EMC), and LiPF_6_(EC:EMC). Scan rate: 50 mV s^–1^.

Figure 3 shows a summary of CV data collected at both the Pt and GC electrodes for the various carbonate electrolytes studied. Measurements were performed by scanning from the open circuit potential to increasingly positive and negative extremes. This allows not only exploration of the limits of the voltage stability window of the electrolyte of choice but also potential assignment of detected products to different stages of the oxidation process. Data collected at varying voltage extremes are shown in different colors to ease comparison and connection to observed products. Figure 3a–c shows voltammograms for Pt in LiPF_6_(EC), LiPF_6_(EMC), and LiPF_6_(EC:EMC), respectively. Initial scans of Pt in LiPF_6_(EC) reveal little indication of electrolyte oxidation up to near 5.0 V (Figure 3a). Scanning above 5.0 V gives rise to an anodic process, while a cathodic current begins to be observed at the lower voltage extreme of the potential window. This cathodic feature continues to become more apparent as the positive voltage limit is increased and eventually leads to the observation of a reversible reduction process that is detected at 3.15 V. Recent RRDE measurements have shown that the observed process is due to reduction of protons generated from the oxidation of the carbonate solvents—a hypothesis that is consistent with computational studies.^28,43,44^ Data from complementary experiments using GC as a working electrode are in Figure 3d–f and show similar oxidation processes, but no reduction product is observed at 3.15 V. This observation is also potentially consistent with the identification of the process at 3.15 V as proton reduction as carbon electrodes are known to have limited reactivity toward this process. Azcarate et al. observed similar anodic decomposition of EC and dimethyl carbonate (DMC) electrolytes.^27^ They found that EC-related species are first oxidized before observing the oxidation process of DMC-related species at nonintercalating electrodes at high voltages. Leung has investigated the mechanisms of EC decomposition on a metal oxide surface with DFT.^45^ Those results indicated that oxidation leads to deprotonation of EC following the initial C–O single bond breakage. In conjunction with the aforementioned experimental and theoretical work, we assign the cathodic process observed at 3.15 V to the reduction of protons generated in the oxidation of the carbonate solvents.

The electrochemical behavior of LiPF_6_(EMC) on a Pt electrode is shown in Figure 3b. The oxidation process of LiPF_6_(EMC) starts showing increased current above 5.0 V, and multiple oxidation processes are once again observed. A large anodic feature at 5.5 V and a cathodic feature at 3.15 V emerge as with the LiPF_6_(EC) system, suggesting that the EMC solvent deprotonates as well during the oxidation process. Zhang et al. explored the oxidation mechanisms of EC-EMC-based electrolytes by in situ Fourier transform infrared spectroscopy (FTIR).^46^ They revealed that the oxidation of carbonate solvents occurs at a high voltage via a deprotonation process. The corresponding deprotonated species (de-H EC and de-H EMC) were identified by FTIR and may participate in further electrochemical processes. To determine the oxidation behavior of the mixed carbonate electrolyte, similar experiments were conducted with Pt in LiPF_6_(EC:EMC). We find similar oxidation processes and reducible products as presented in Figure 3c. This suggests that the oxidation process of EC:EMC-based electrolytes likely also proceeds through deprotonation of both EC and EMC. The results for oxidation at a GC electrode shown in Figure 3d–f lack the reduction product at 3.15 V but do better resolve multiple overlapping oxidation processes that appear to be occurring for all three electrolytes studied, indicating varied oxidation reactivity between the two electrode surfaces.

In summary, our CV results clearly show that oxidation of our varied electrolytes leads to the detection of a quasireversible reduction process at 3.15 V, potentially due to deprotonation of the involved carbonate solvents. However, these results do not allow us to connect the formation of the product at 3.15 V to any of the specific oxidation processes seen on either the Pt or the GC electrode surfaces.

**Figure 4:**
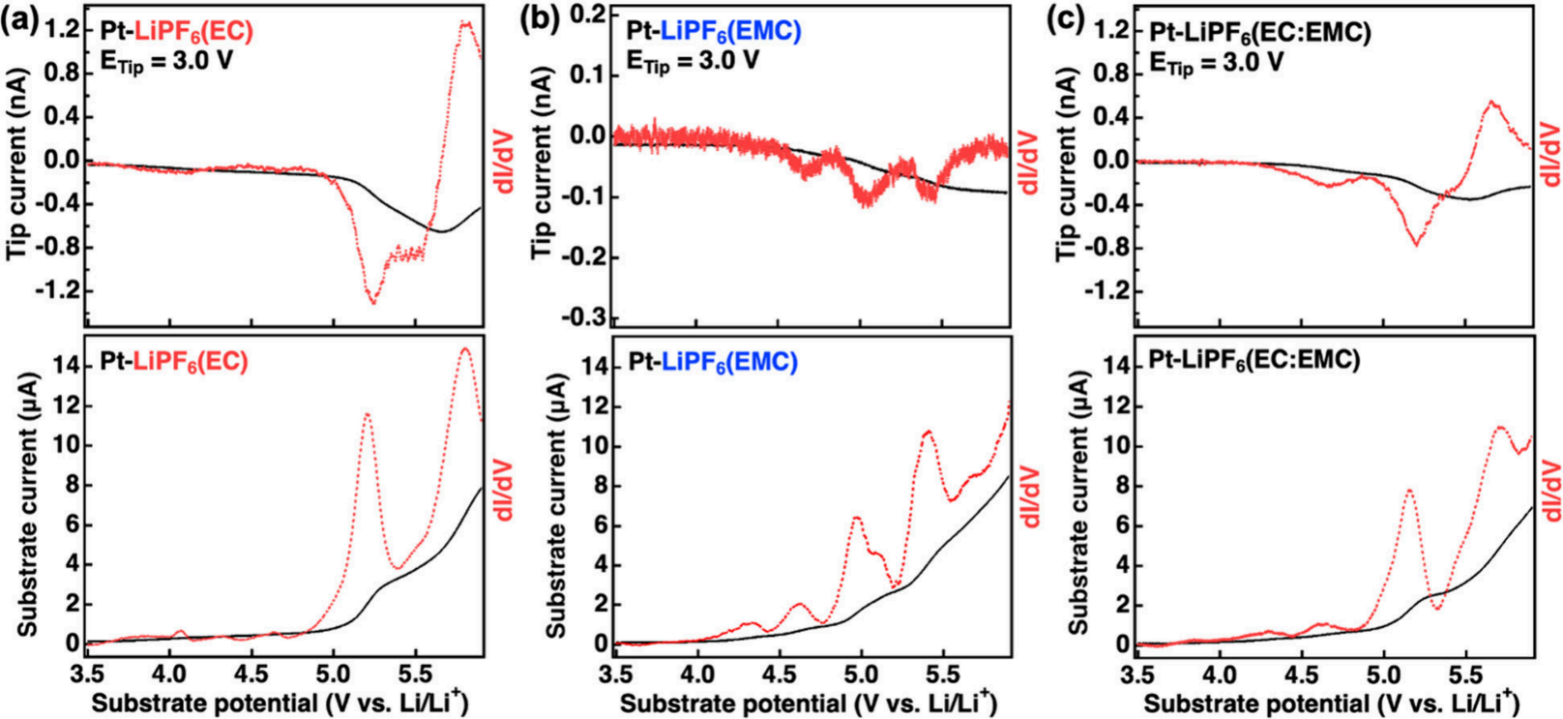
G/C SECM measurements for a Pt electrode (2 mm) with a UME Pt tip (10 μm) at 3.0 V in (a) LiPF_6_(EC), (b) LiPF_6_(EMC), and (c) LiPF_6_(EC:EMC). Black solid traces show tip/substrate current collected in SECM measurements, while red dashed traces show data in a differential (d*I*/d*V*) format.

For comparison to the voltammetry results discussed above, G/C SECM measurements were conducted by using a 2 mm Pt electrode as a substrate and a 10 μm Pt electrode as the probe tip. Tip positioning was accomplished as described previously. As in earlier experiments, the substrate voltage was swept from 3.5 to 6.0 V to drive the oxidation of the relevant electrolyte, while the tip was biased either at 3.0 or 3.7 V (Figure 4 and S3). At 3.0 V, the tip is biased sufficiently negative to allow detection of the product reduced at 3.15 V (if present). At 3.7 V, the tip cannot reduce the product detected at 3.15 V but may detect alternate products that may be reducible at more positive voltages. Comparison of these two data sets can help to connect the 3.15 V reduction process to the different oxidation processes occurring at the substrate. In Figures 4 and S3, the lower plots show LSV data for the oxidation of the electrolyte at the substrate electrode, while tip currents are shown in the upper plots. To ease the identification of overlapping processes in the *I*–*V* curves, data are presented both as current (solid traces) and in a differential (d*I*/d*V*) format (dashed traces). The d*I*/d*V* data were calculated as described in the Experimental Section and allow inflection points (potentially indicative of distinct oxidation processes) in the current data to be more readily recognizable.

Figure 4a shows the characterization of LiPF_6_(EC) oxidation when scanning the Pt substrate from 3.5 to 6.0 V while holding the tip at 3.0 V. The substrate current (lower plot) shows an increase in oxidation current just above 5.0 V then followed by a more gradual increase up to 6.0 V similar to the trend observed in the comparative CV measurements. The d*I*/d*V* plots indicate what appear to be two distinct oxidation processes occurring at ∼5.1 and ∼5.7 V. As shown in Figure S3a, the tip current collected at 3.7 V shows no detectable oxidation products, while that collected at 3.0 V clearly shows increasing current that coincides with substrate oxidation processes. This data are consistent with earlier CV results and indicate that the detected products may be connected to the deprotonation reaction. The tip current collected at 3.0 V shows an increase upon the initiation of electrolyte oxidation at the substrate at ∼5.0 V. This indicates that this process generates a soluble, reducible product, which is likely linked to the deprotonation of EC. The tip current continues to increase until reaching a maximum when scanning the substrate to ∼5.7 V and then decreases. Comparison of the d*I*/d*V* traces for substrate and tip data appears to show that the initial oxidation process at the substrate observed at ∼5.1 V leads to a reducible product detected at the tip; however, the second oxidation process occurring at ∼5.7 V results in a subsequent decrease in tip current. It is reasonable that detection of the reduction product at 3.15 V would yield a steady state response at the tip through an SECM feedback process given the quasireversible nature of the CV data shown in Figure 2. Given this, we believe that the first process detected for oxidation of EC is the same as that detected at 3.15 V in CV data. However, the decrease in tip current coincident with the start of the second oxidation process at the substrate appears to indicate a disruption of the feedback process. This decrease in tip current may occur due to removal of electroactive products from the gap between the tip and substrate through chemical or electrochemical reactions or potentially passivating film formation on the tip or substrate surfaces.

It has been reported that the decomposition of carbonate electrolytes will lead to the formation of an organic-species-rich film on GC electrode surfaces.^27^ The films are not stable and appear to be soluble at low voltage but become stable and passivating when the electrode was held at high voltage; therefore, film formation is possible. However, our attempts to detect film presence electrochemically or spectroscopically have shown no definitive evidence of film formation. This appears to indicate that the origin of the decreased tip current may be the chemical/electrochemical reactivity of the products of the second oxidation through solution phase reactions potentially including the products of the first oxidation process.

Figure 4b shows the same G/C SECM data collected for LiPF_6_(EMC). The observed substrate current showed a more gradual increase in current with increasing voltage compared to EC. Interestingly, analysis of the d*I*/d*V* data reveals many more anodic features, with potentially as many as eight separate processes detected as compared to the two seen for EC-based electrolytes. Tip current collected at 3.7 V showed a small increase in tip current was observed when the substrate voltage was above 5.5 V (Figure S3b). This indicates that at high voltage the EMC-based electrolyte potentially forms a different product than the EC-based electrolyte and that this species is reduced at voltages greater than 3.7 V. That product was not easily identifiable by earlier CV measurements and may indicate the formation of a species at extreme voltages for the EMC-based electrolyte that is detectable on the time scale of the G/C SECM experiment (∼203 ms) but may not be stable in the time scale of the earlier voltametric analysis (∼37 s). Tip current collected at 3.0 V shows electroactive products formed from at least three of the oxidation processes observed on the substrate. Substrate oxidation processes occurring at around 4.6, 5.0, and 5.4 V appear to yield products which can be reduced at 3.0 V (Figure 4b). Additional oxidation steps appearing in the substrate current are apparently not directly related to the formation of soluble electroactive products and may be more related to film formation or the generation of nonelectroactive species. Notably, the *I*–*V* traces collected from the tip at 3.0 V do not show the same decrease that was observed at high voltage for the oxidation of EC but lead instead to a series of sigmoidally shaped responses similar to that observed for the Fc/Fc^+^ model system. This potentially indicates that the products formed during oxidation of EMC can cycle between reduction and oxidation at the tip and substrate electrodes indicating a greater relative stability in solution than the product of the second oxidation process observed for EC.

Finally, Figure 4c shows data for the LP58 electrolyte, which contains a blend of 30% EC and 70% EMC (wt %). Substrate currents show multiple oxidation processes occurring which are similar to those seen from both EC and EMC alone including the anodic features at 5.1 and 5.7 V. Again, tip current collected at 3.7 V showed no detectable products at low voltage; however, the same slight increase in tip current seen for EMC oxidation was observed above 5.5 V (Figure S3c). Tip data collected at 3.0 V showed several processes that are similar to those seen for EMC and EC; however, not all of the same processes are as evident. Substrate data shows oxidation processes at around 4.3, 4.6, 5.1, and 5.7 V. The initial oxidation processes showing below 5.0 V appear to be the same seen for the EMC-based electrolyte, while processes at higher voltage appear to be dominated by oxidation of EC. A similar decrease in tip current at high voltage as was observed for EC alone is also observed here. These observations imply that the electrochemical oxidation of mixed carbonates may not behave the same as the single carbonates. Similar results were reported in the study on the oxidation behavior of LP30 (EC:DMC) electrolytes.^27^

In summary, our analysis reveals that the oxidation of the LP58 electrolyte begins with an initial oxidation of EMC-related species with features observed at 4.3 and 4.6 V. It appears that the oxidation at 4.3 V does not produce a soluble, reducible product, while the second oxidation does. At higher voltages, the oxidation current appears to be dominated by EC-related species with processes occurring at 5.1 and 5.7 V. The initial oxidation at 5.1 V leads to formation of a soluble, reducible product, while the second process at 5.7 V seems to shut down the “feedback” process occurring between the tip and substrate electrodes. This potentially indicates that the second oxidation may lead to consumption of the initial oxidation product through chemical or electrochemical reactions or through the formation of passivating films on either the substrate or tip electrodes. Similar data were also collected using a GC substrate and yielded similar results to those presented here. These results are shown and discussed in the supporting information (Figures S4 and S5).

**Figure 5:**
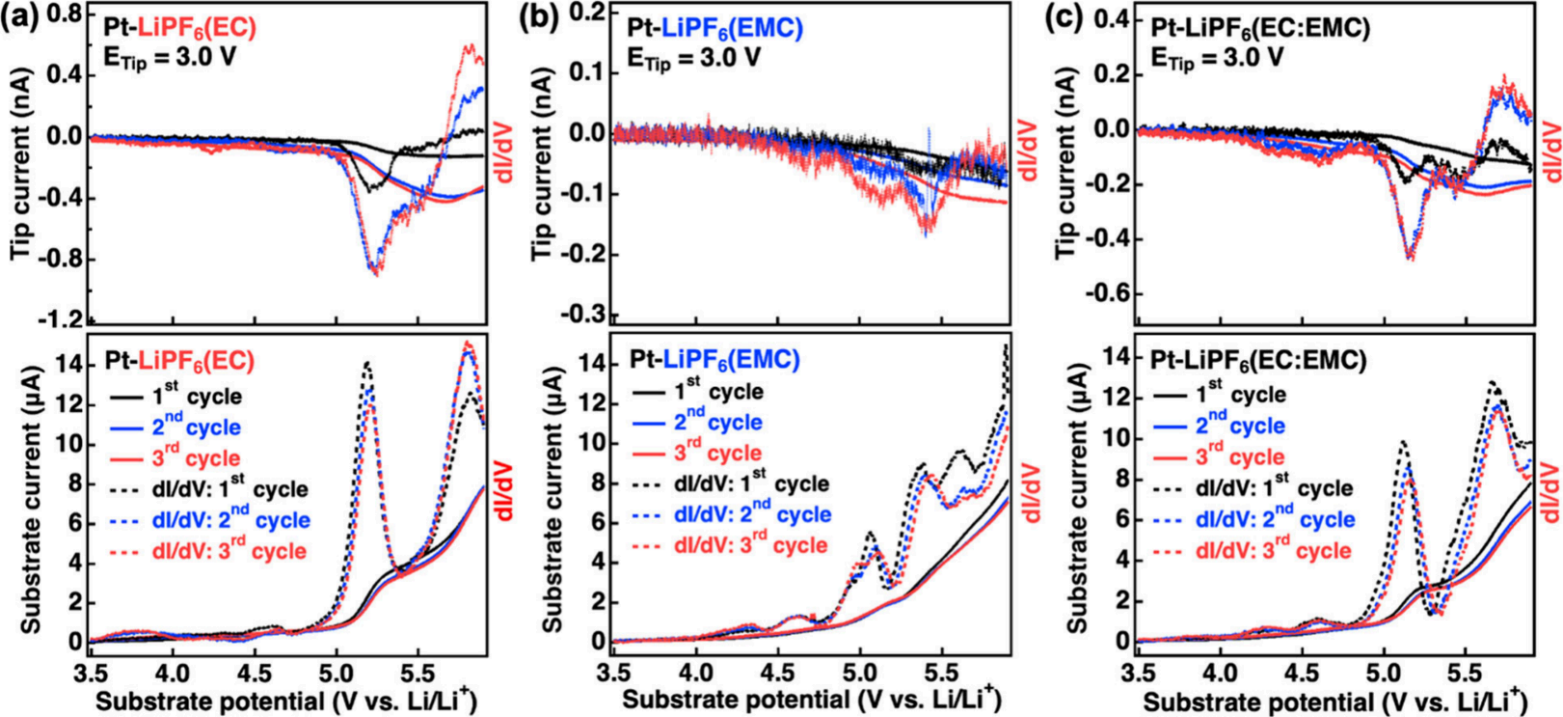
G/C SECM measurements for a Pt electrode (2 mm) with a UME Pt tip (10 μm) at 3.0 V in (a) LiPF_6_(EC), (b) LiPF_6_(EMC), and (c) LiPF_6_(EC:EMC) with repeated cycling. Three cycles are performed in a row. Solid curves represent the substrate and tip currents, and associated d*I*/d*V* profiles are presented in a dashed format.

CEI processes and films are believed to evolve throughout cycling, as discussed earlier. Based on this, we examined how the observed G/C SECM voltammetry results evolved as a function of cycles of oxidation and the data collected with a Pt disk electrode are summarized in Figure 5. In this experiment, the tip positioning was accomplished as before, and the substrate voltage was swept from 3.5 to 6.0 V, while the tip current was monitored at 3.0 V as in Figure 4. The black trace shows data for the first LSV sweep of the substrate; blue shows the second, and red shows the third. As before, the data are presented in a d*I*/d*V* format to ease observation of differing oxidation processes. As shown in Figure 5a–c, the first three cycles of G/C SECM measurements were recorded for LiPF_6_(EC), LiPF_6_(EMC), and LiPF_6_(EC:EMC), respectively. Substrate oxidation traces show a response similar to that shown in Figure 4a–c. However, some differences are noted; in particular, the reduction current detected at the tip for cycles 2 and 3 is significantly higher than that observed in cycle 1, although only minor changes appear to occur in the substrate currents. Of particular interest is the EMC oxidation process detected at ∼5.6 V, which is observed in the first cycle but is greatly attenuated in cycles 2 and 3. It is also noted that this process does not appear to lead to an appreciable reduction signal at the tip electrode. This may tie that reaction to formation of either a nonelectroactive product or potentially a film-forming process at the substrate that only occurs within the first cycle. The increased tip current in the second and third cycles may indicate that the substrate surface has been activated toward further electrolyte oxidation after the initial cycle. Similar results were obtained with a GC electrode in G/C SECM cycling measurements (Figure S6).

## Conclusions

In summary, we have developed and demonstrated generation/collection SECM-based characterization methods focused on the oxidation behavior of varied carbonate-based electrolytes containing the LiPF_6_ salt on nonintercalating Pt/GC substrates. We have compared the G/C SECM method to CV studies of oxidation processes for the same electrolytes. CV analysis shows that oxidation of electrolytes containing only the EC and EMC solvents as well as the blended LP58 electrolyte leads to the detection of a reducible product observed at 3.15 V that is potentially associated with deprotonation of the carbonate solvents. G/C SECM results show similar oxidation processes as CV but also show that only some of those oxidation processes appear to be connected to deprotonation of the carbonates, while others may form other products. G/C SECM results show that oxidation of LiPF_6_(EC) appears to consist of at least two different processes. The first process appears to be related to deprotonation of EC, while the second oxidation is likely a different process, which appears to impede the earlier observed proton reduction process. This may occur through consumption of the proton or through film formation that may impede detection by the tip or substrate. Oxidation of LiPF_6_(EMC) may entail up to eight different detected processes. Only three of these appear to be due to the formation of a reducible product detected at 3.0 V and are potentially due to deprotonation reactions or coupled processes. Oxidation (and concomitant reduction) currents for EMC were lower than that measured for EC, potentially indicating less activity toward oxidation. The blended carbonate electrolyte (LP58) showed oxidation signatures consistent with both the EC and EMC. Oxidation processes occurring at lower voltages appear to be related to EMC, while the higher voltage response for the blend appeared to be dominated by oxidation of EC. Our data is consistent with the oxidation of the carbonate electrolytes involving many steps, some of which yield soluble, electroactive products, while others may yield either nonelectroactive products or potential film-forming products. These results demonstrate new perspectives for electrolyte decomposition and novel approaches to detect oxidation products. Our findings are expected to provide a reference for future design of more stable electrode-electrolyte interfaces.

## Supporting Information Available

SECM approach curve process; G/C SECM measurements of GC electrode; performance comparison, and supplementary discussion (PDF)
