# Supplementary material for: In Situ Characterization of the Oxidation Behavior of Carbonate-Based Electrolytes for Lithium-Ion Batteries by Scanning Electrochemical Microscopy
Source: ACS Electrochem. 2024 Dec 4;1(4):494–503. doi: 10.1021/acselectrochem.4c00106 (PMC11973861; doi:10.1021/acselectrochem.4c00106)
Supplement: Supplementary file 1 — ec4c00106_si_001.pdf [file ec4c00106_si_001.pdf]

Supporting Information

In Situ Characterization of the Oxidation Behavior of Carbonate-Based Electrolytes for Lithium-Ion Batteries by Scanning Electrochemical Microscopy

Rong He,[1,2] Liam McDonough,[1] Liam Seitz,[1] Wenhan Ou,[3] Samuel D. Marks,[3] Rafael Ferreira de Menezes,[4] Elizabeth Allan-Cole,[4] Hongmei Luo,[2] Michael F. Toney,[3,4,5] Kayla G. Sprenger,[4] Meng Zhou,[2,*] and Robert C. Tenent[1,5,*]

[1] National Renewable Energy Laboratory, Golden, CO 80401, United States.

E-mail: *robert.tenent@nrel.gov*

[2] Department of Chemical and Materials Engineering, New Mexico State University, Las Cruces, NM 88003, United States. E-mail: *mzhou@nmsu.edu*

[3] Materials Science and Engineering, University of Colorado Boulder, Boulder, CO 80303, United States.

[4] Department of Chemical and Biological Engineering, University of Colorado Boulder, Boulder, CO 80303, United States.

[5] Renewable and Sustainable Energy Institute, University of Colorado at Boulder, Boulder, CO 80309, United States.

Table of contents

Figure S1. Cyclic voltammetry of a UME Pt tip (10 μm) in $LiPF_6$(EC:EMC)-Fc electrolyte before and after rinsing with $LiPF_6$(EC:EMC) electrolyte.

Figure S2. A probe approach curve of a UME Pt tip (10 μm) in $LiPF_6$(EC:EMC)-Fc electrolyte over a 2 mm Pt disk substrate electrode.

Figure S3. G/C SECM measurements for a Pt electrode (2 mm) with a UME Pt tip (10 μm) at 3.7 V in (a) $LiPF_6$(EC), (b) $LiPF_6$(EMC), and (c) $LiPF_6$(EC:EMC).

Figure S4. G/C SECM measurements for a GC electrode (3 mm) with a UME Pt tip (10 μm) at 3.0 V in (a) $LiPF_6$(EC), (b) $LiPF_6$(EMC), and (c) $LiPF_6$(EC:EMC).

Figure S5. G/C SECM measurements for a GC electrode (3 mm) with a UME Pt tip (10 μm) at 3.7 V in (a) $LiPF_6$(EC), (b) $LiPF_6$(EMC), and (c) $LiPF_6$(EC:EMC).

Figure S6. G/C SECM measurements for a GC electrode (3 mm) with a UME Pt tip (10 μm) at 3.0 V in (a) $LiPF_6$(EC), (b) $LiPF_6$(EMC), and (c) $LiPF_6$(EC:EMC) with repeated cycling.

SECM Approach Curve Process. Scanning electrochemical microscopy (SECM) measurements were performed by positioning a ultramicroelectrode (UME) Pt tip near the substrate surface. Prior to tip approach, cyclic voltammetry (CV) was used to confirm appropriate electrochemical behavior of the Pt tip in the redox mediator (ferrocene/ferrocenium, Fc/Fc^{+}) solution. The red trace in **Figure S1**, shows the oxidation and reduction of ~0.3 mM Fc in the LP58 electrolyte. A curve indicating the oxidation and reduction of the Fc redox mediator is observed at ~3.2 V. Tip positioning was achieved by applying the probe approach curve (PAC) technique with a positive feedback mode. An example approach curve is shown in **Figure S2**. A voltage of 3.4 V is applied to the tip, allowing ferrocene (Fc) to be oxidized to ferrocenium (Fc^{+}) while the substrate voltage is set to 3.0 V to reduce Fc^{+}. Tip approach was stopped when the tip current increased to 125% of that observed in bulk solution. For a 10 μm dia. Pt disk tip the distance between tip and substrate is calculated to be ~ 10.5 μm. From calculations based on a measured diffusion coefficient of 2.72E-6 cm^2/s for ferrocene in the $LiPF_6$(EC:EMC) electrolyte we estimate a transit time of ~ 203 ms from generation at the substrate to detection of the tip at these distances. Following tip positioning, the $LiPF_6$(EC:EMC)-Fc electrolyte was removed, and the cell was rinsed several times with fresh $LiPF_6$(EC:EMC) electrolyte before further SECM measurements. A second tip CV was collected after rinsing the cell and is shown as the blue trace in **Figure S1**. This shows that no detectable Fc was remaining in the cell after rinsing. The same procedure was applied when conducting all measurements presented.

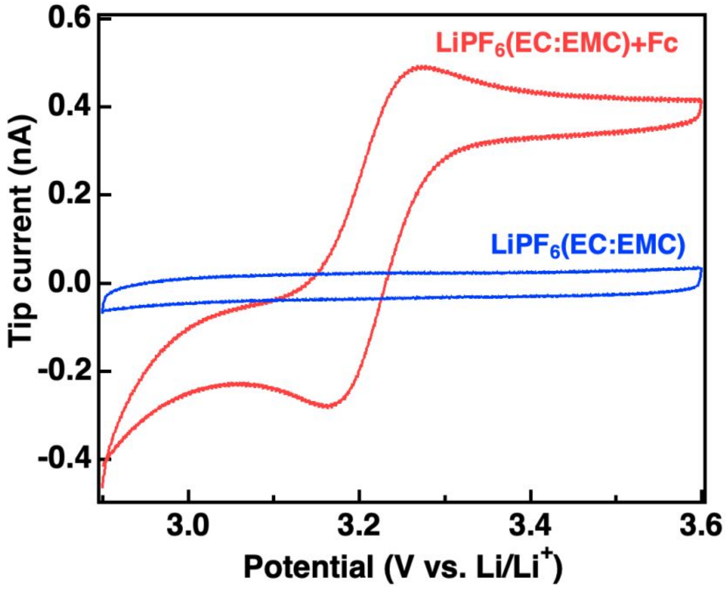

Figure S1. Cyclic voltammetry of a UME Pt tip (10 μm) in $LiPF_6$(EC:EMC)-Fc electrolyte before and after rinsing with $LiPF_6$(EC:EMC) electrolyte.

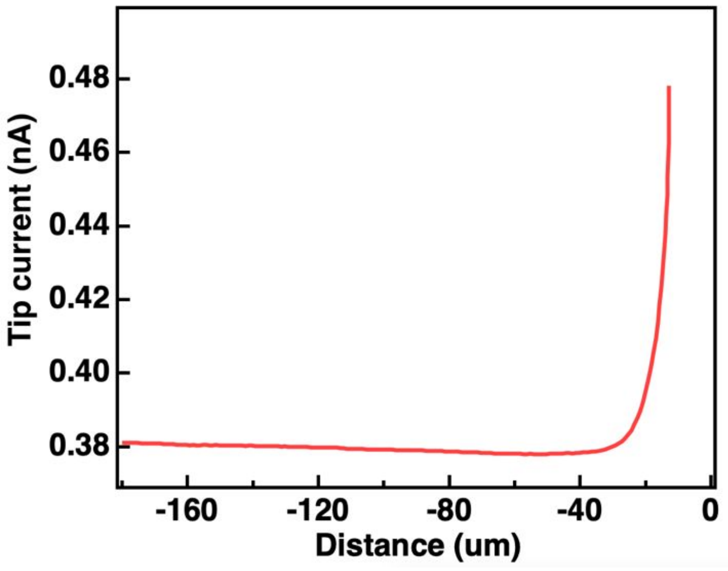

Figure S2. A probe approach curve of a UME Pt tip (10 μm) in $LiPF_6$(EC:EMC)-Fc electrolyte over a 2 mm Pt disk substrate electrode. E_{sub} = 3.0 V; E_{tip} = 3.4 V.

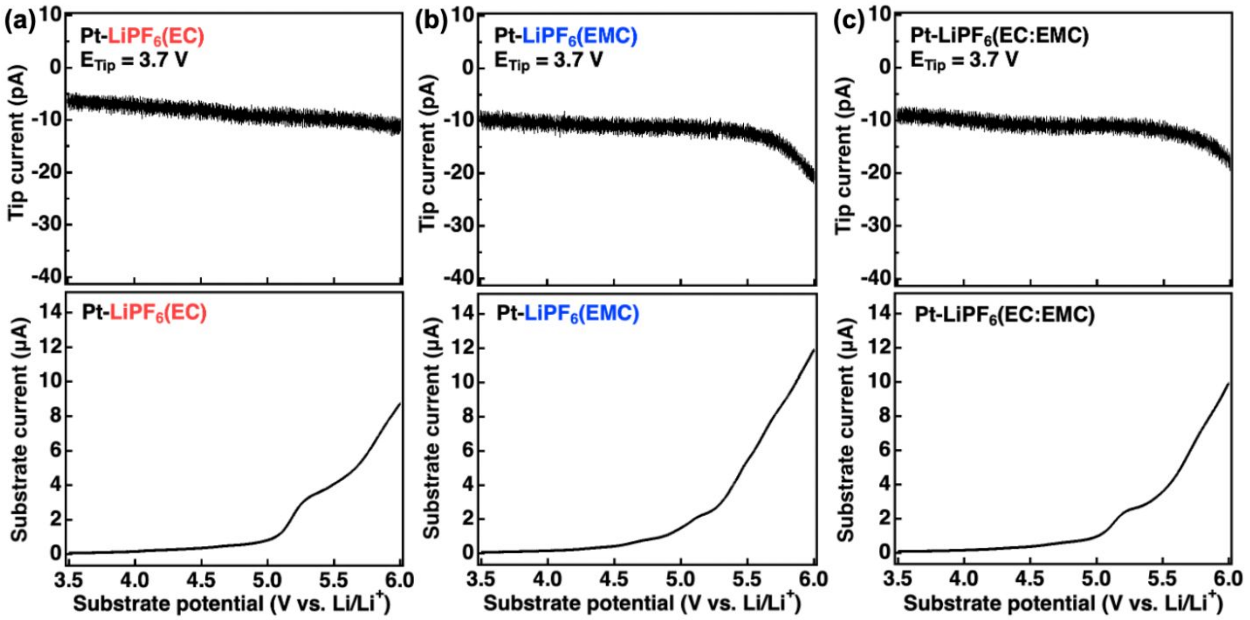

Figure S3. G/C SECM measurements for a Pt electrode (2 mm) with a UME Pt tip (10 μm) at 3.7 V in (a) $LiPF_6$(EC), (b) $LiPF_6$(EMC), and (c) $LiPF_6$(EC:EMC).

G/C SECM measurements at a GC electrode. Electrolyte decomposition was explored using G/C SECM with a non-intercalating GC electrode and a 10 μm Pt tip. Data is presented both as current vs. voltage and in differential form as in the main manuscript. As shown in **Figure S4**, multiple anodic features are observed in the LSV scans (black curve) for the GC substrate in $LiPF_6$(EC), $LiPF_6$(EMC), and $LiPF_6$(EC:EMC) When applying a voltage of 3.0 V to the tip, soluble and reducible products were detected that coincided with oxidation processes at the GC substrate above high voltage (> 5.0 V). No significant tip signal was detected when the tip voltage was held at 3.7 V (**Figure S5**), which indicates that the oxidation process for EMC above 5.5 V observed on a Pt substrate may not occur on GC. Tip current data collected at 3.0 V clearly shows that multiple oxidation processes at GC generate soluble, reducible species. These results are qualitatively similar to those seen with the Pt substrate. It is worth noting that while earlier CV results of the GC electrode are consistent with similar oxidation processes occurring on both Pt

and GC, the return reduction sweeps did not detect the same product shown at 3.15 V for Pt. As stated in the main manuscript this is consistent with the species at 3.15 V likely being due to deprotonation of the carbonate solvents. The fact that the oxidation products from the GC substrate generates a species which can be detected at the Pt tip electrode when using the G/C SECM technique further corroborates the likelihood that similar oxidation processes are occurring and products being formed on the GC substrate as those seen on Pt. Results for the oxidation $LiPF_6$(EC) are shown in **Figure S4a** and are similar to those seen for the Pt substrate with two oxidation processes being observed at the substrate at 5.0 V and 5.5 V. This is a slight shift from that observed for oxidation on Pt. Examination of the tip current collected at 3.0 V shows that the initial oxidation leads to detection of the soluble, reducible product at the tip while the second oxidation process leads to a decrease in tip current. This is the same as was observed for the Pt experiments and potentially indicates that the second oxidation either consumes the products of the first or may shut down feedback between the tip and substrate through film formation. **Figure S4b** shows data for the oxidation of $LiPF_6$(EMC). Similar to earlier results for oxidation on Pt shown in the main manuscript for EMC(**Figure 4b**), a lower overall current is detected for the EMC oxidation and concomitant reduction processes at the tip. Again, multiple oxidation processes are observed for EMC on GC, however fewer clear reduction products are detected. This may indicate that the oxidation processes observed change between Pt and GC electrodes leading the formation of fewer soluble reducible products but may also indicate that formed products interact with the GC substrate differently. **Figure S4c** shows data for the LP58 blended electrolyte $LiPF_6$(EC:EMC). Once again, a similar response to that seen for Pt in **Figure 4c** is observed for GC and appears to be consistent with the oxidation of EMC related species occurring at lower voltage while higher

voltage processes are dominated by EC oxidation. The same decrease in tip current occurs following the second EC related oxidation as was seen previously for EC alone.

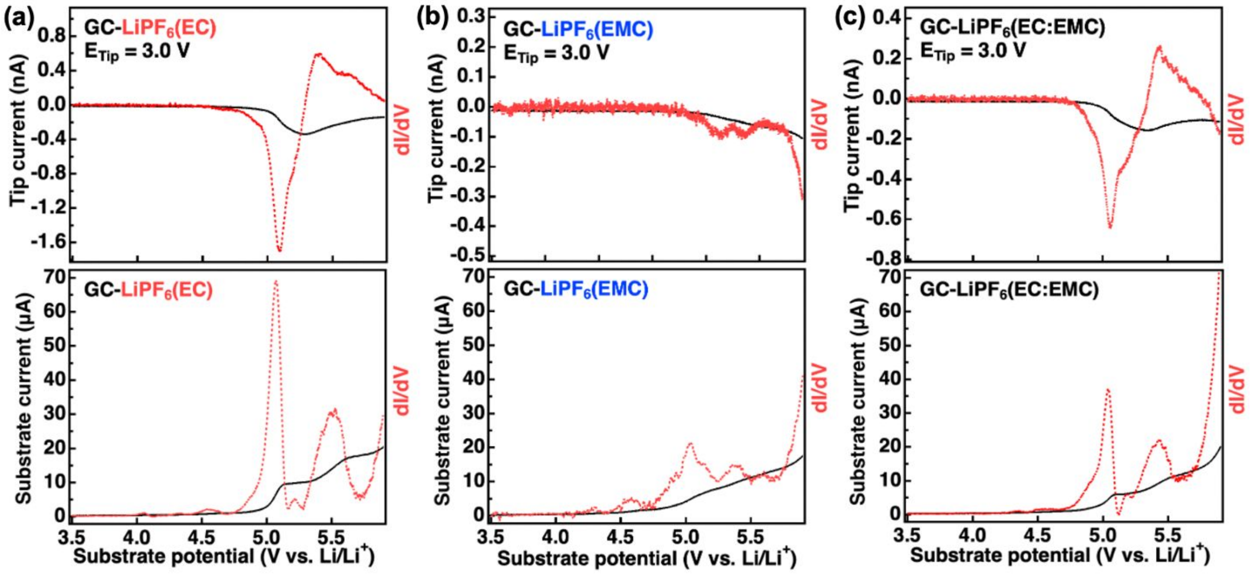

Figure S4. G/C SECM measurements for a GC electrode (3 mm) with a UME Pt tip (10 μm) at 3.0 V in (a) $LiPF_6$(EC), (b) $LiPF_6$(EMC), and (c) $LiPF_6$(EC:EMC). Black solid traces show tip/substrate current collected in SECM measurements while red dashed traces show data in a differential (dI/dV) format.

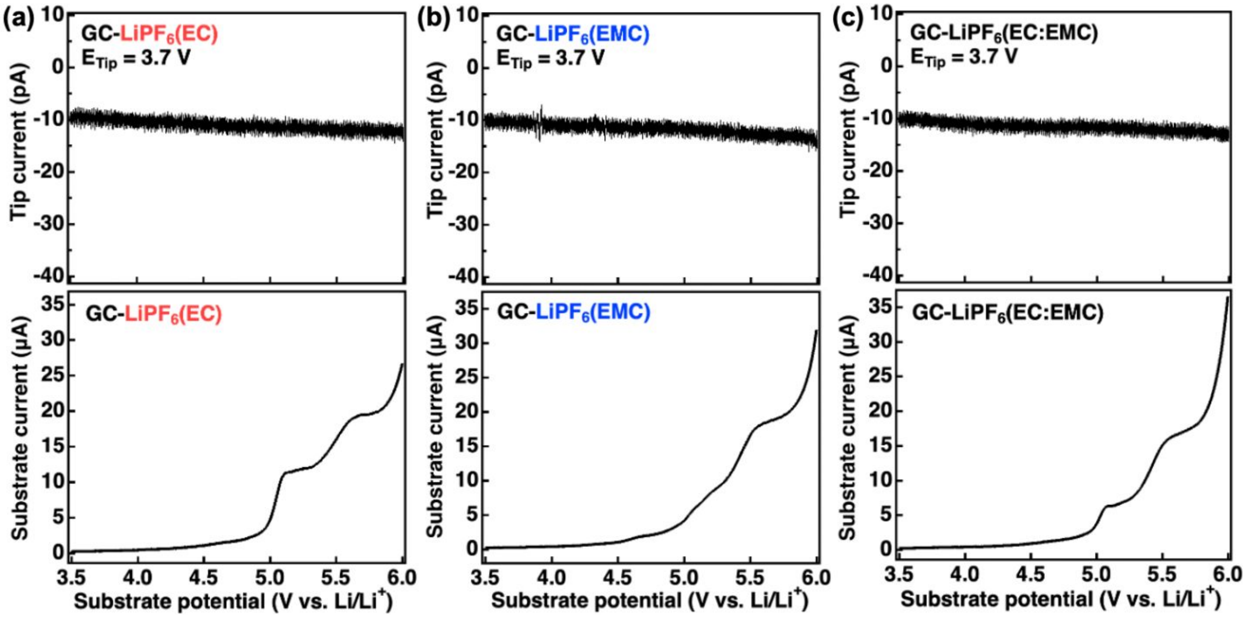

Figure S5. G/C SECM measurements for a GC electrode (3 mm) with a UME Pt tip (10 μm) at 3.7 V in (a) $LiPF_6$(EC), (b) $LiPF_6$(EMC), and (c) $LiPF_6$(EC:EMC).

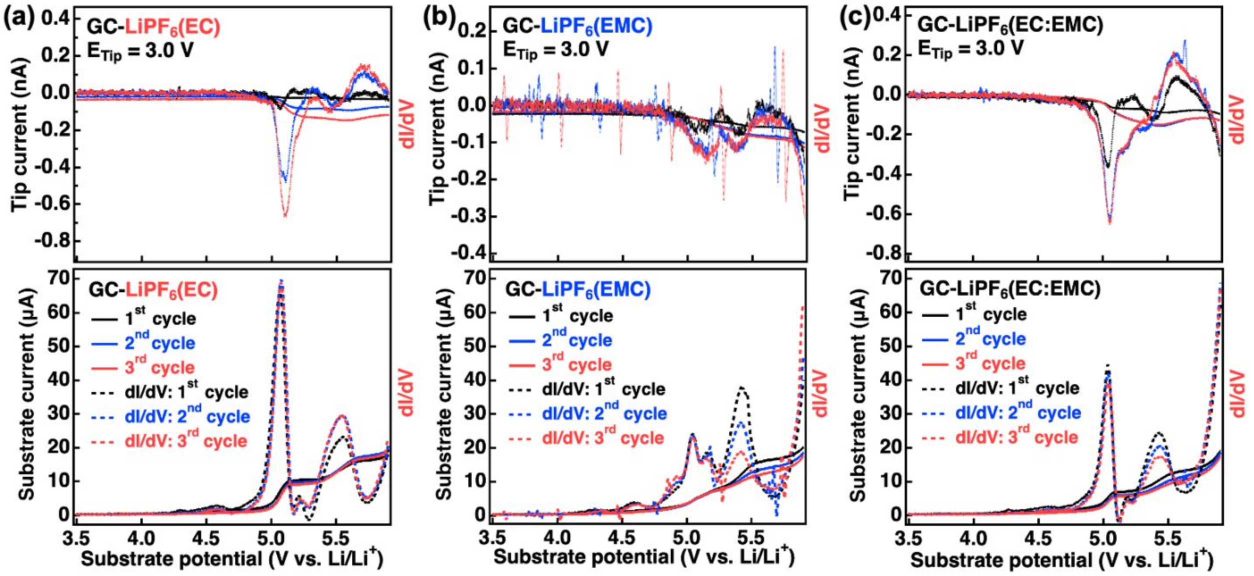

Figure S6. G/C SECM measurements for a GC electrode (3 mm) with a UME Pt tip (10 μm) at 3.0 V in (a) $LiPF_6$(EC), (b) $LiPF_6$(EMC), and (c) $LiPF_6$(EC:EMC) with repeated cycling. 3 cycles are performed in a row. Solid curves represent the substrate and tip currents and associated dI/dV profiles are presented in a dashed format.
